# Temperature-dependent tensile properties of polyamide 12 for the use in percutaneous transluminal coronary angioplasty balloon catheters

**DOI:** 10.1186/s12938-021-00947-8

**Published:** 2021-10-26

**Authors:** C. Amstutz, B. Weisse, S. Valet, A. Haeberlin, J. Burger, A. Zurbuchen

**Affiliations:** 1 Sitem Center for Translational Medicine and Biomechanical Entrepreneurship, University of Bern, Freiburgstrasse 3, 3010 Bern, Switzerland; 2 EMPA, Swiss Federal Laboratories for Material Science and Technology: Mechanical Systems Engineering, Duebendorf, Switzerland; 3 Present Address: Department of Cardiology, Inselspital, Bern University Hospital, University of Bern, Bern, Switzerland

**Keywords:** PA 12, Mechanical properties, Necking, PTCA balloon catheter, Tensile testing

## Abstract

**Background:**

Percutaneous transluminal coronary angioplasty (PTCA) balloon catheters must withstand high pressures required for the lesion treatment, pushing loads during insertion, and pulling loads during withdrawal. These loads pose a challenge especially for polymeric tubular shafts with small cross sections. In order to enable new design innovations and to better understand the mechanics of current catheter technologies, the tensile properties of polyamide (PA) 12 were investigated. PA 12 dog bone specimens and medical PA 12 tubes were either stored at ambient temperature and humidity or conditioned in water, and subjected to tensile loads at different temperatures. In addition, the effect on the tensile properties of the necking process, a forming process to reduce the wall thickness of the tubes, was determined.

**Results:**

The tested tubes showed a reduction in both Young’s Modulus (− 41.5%) and yield stress (− 29.2%) compared to standardized specimens. Furthermore, an increase in temperature and water absorption softens the material and reduces the mechanical properties like the Young’s Modulus and the yield stress. It was found that the material strengthens during the necking process. Likely due to the orientation of the polymers chain molecules in load direction (Rösler et al., 2007), the Young’s Modulus of the material could be increased by 43.5%. Furthermore, the absence of a yield point after necking allows for a greater loading capacity of the material without unstable neck growth. Besides the strengthening, the ultimate strain is reduced by 50%. This indicates that the necking process induces plastic deformation.

**Conclusion:**

The investigation showed that the environmental conditions like temperature and humidity can influence mechanical properties. It could also be shown that pre-forming processes such as necking can enhance the mechanical properties, such as the Young’s Modulus, while reducing the wall thickness. These findings suggest possible further development of catheters with a small cross section and higher mechanical strength and highlight the importance to account for the targeted operating temperature during the design process.

## Background

Polymers are widely used in medical devices [1] and becoming increasingly important. Polymers offer specific properties, such as a high compliance, low weight, good biocompatibility, as well as allow building affordable single-use devices such as percutaneous transluminal coronary angioplasty (PTCA) balloon catheters.

PTCA balloon catheters are used to restore the blood flow at occluded or narrowed (stenotic) coronary arteries. Current demographic changes promote cardiovascular risk factors like hypertension and obesity. Such factors favor calcified and resistant stenoses, which in return increase the mechanical loads on PTCA balloon catheters [2], and at the same time, require smaller cross sections for treatment of narrow stenoses.

During a minimal-invasive procedure, a balloon catheter is advanced with the help of a guide wire to the stenotic region and inflated to open narrowed or blocked arteries [3]. In order to maneuver the balloon to the stenotic region, a PTCA balloon catheter must combine different mechanical properties. The proximal section (hypotube) of the catheter is usually made of a stiff metallic tube to provide sufficient pushability, which enables the delivery of the catheter to the coronary arteries. The distal section, on the other hand, is made from more flexible polymeric tubes that can follow tortuous vessels without harming them. At the site of the stenosis, the balloon is inflated to a defined pressure, that can reach up to 35 atm depending on the balloon type. Therefore, the catheter must withstand high pressures required for the inflation, pushing loads during insertion, and pulling loads during withdrawal. In combination with a small cross section, these loads impose a challenge especially to the polymer shaft.

In addition to the polymers such as polyethylene terephthalate (PET) or polyether block amide (PEBA), polyamide (PA) 12 is typically used for the manufacturing of distal PTCA balloon catheter shafts. The semi-crystalline polymer PA 12 is formed by polymerization of a 12-carbon atom monomer, laurolactam (lactam 12).

PA 12 does not only demonstrate both high strength, high compliance, and stress crack resistance, but also exhibits a good chemical and abrasions resistance, as well as a low coefficient of sliding friction [4]. It is already known that the properties of polyamide can be affected by humidity or a watery environment [5]. Unlike other polyamides, the low concentration of amide groups in PA 12 promotes a low moisture absorption (≈ 0.7–2%).

The mechanical properties of polymers do not only depend on the polymer itself, but also on the environment (e.g., surrounding temperature and media), the usage (e.g., long-, or short-term, loading velocity) and the manufacturing technology used to produce the device [6]. Although PA 12 is widely used, so far not a lot of mechanical properties have been published. Brydson [7], McKeen and Massey [4], as well as manufacturers like EMS-GRIVORY [8], published some general mechanical properties. Past studies also focused on the manufacturing of PA 12 angioplasty balloons [9, 10]. Recently, Geith [11] published for the first time the anisotropic mechanical behavior of a PA 12 balloon catheter membrane. However, to the author's knowledge no publication yet showed the influence of the temperature, water absorption and material processing methods on mechanical properties of PA 12. Since PA 12 is a commonly used material for PTCA balloon catheter, a better understanding of the influences on the mechanical properties facilitates development of the medical device. Therefore, the aim of this study was to investigate the influence of the temperature, water absorption, sample geometry, and forming steps such as necking, on the tensile properties of PA 12 for PTCA balloon catheter shafts.

## Results

The results of the differential scanning calorimetry (DSC) can be seen in Table 1. No significant deviation between the groups was observed. The crystallinity was for all three specimens around 25.7 ± 0.53%. The glass transition temperature ${T}_{\mathrm{g}}$ and the melting temperature ${T}_{\mathrm{m}}$ was found to be at 52.7 ± 4.35 °C and at 178.7 °C, respectively.

**Table 1:** Results of the DSC test on one specimen of the dog bone, tube, and the necked tube

Properties	Unit	Dog bone	Tube	Necked tube
${T}_{\mathrm{m}}$	°C	179.7	178.3	178.1
Crystallinity	%	26.2	26.0	25.6
${T}_{\mathrm{G}}$	°C	46.4	56.4	54.4

### Results from the dog bone test

**Fig. 1:**
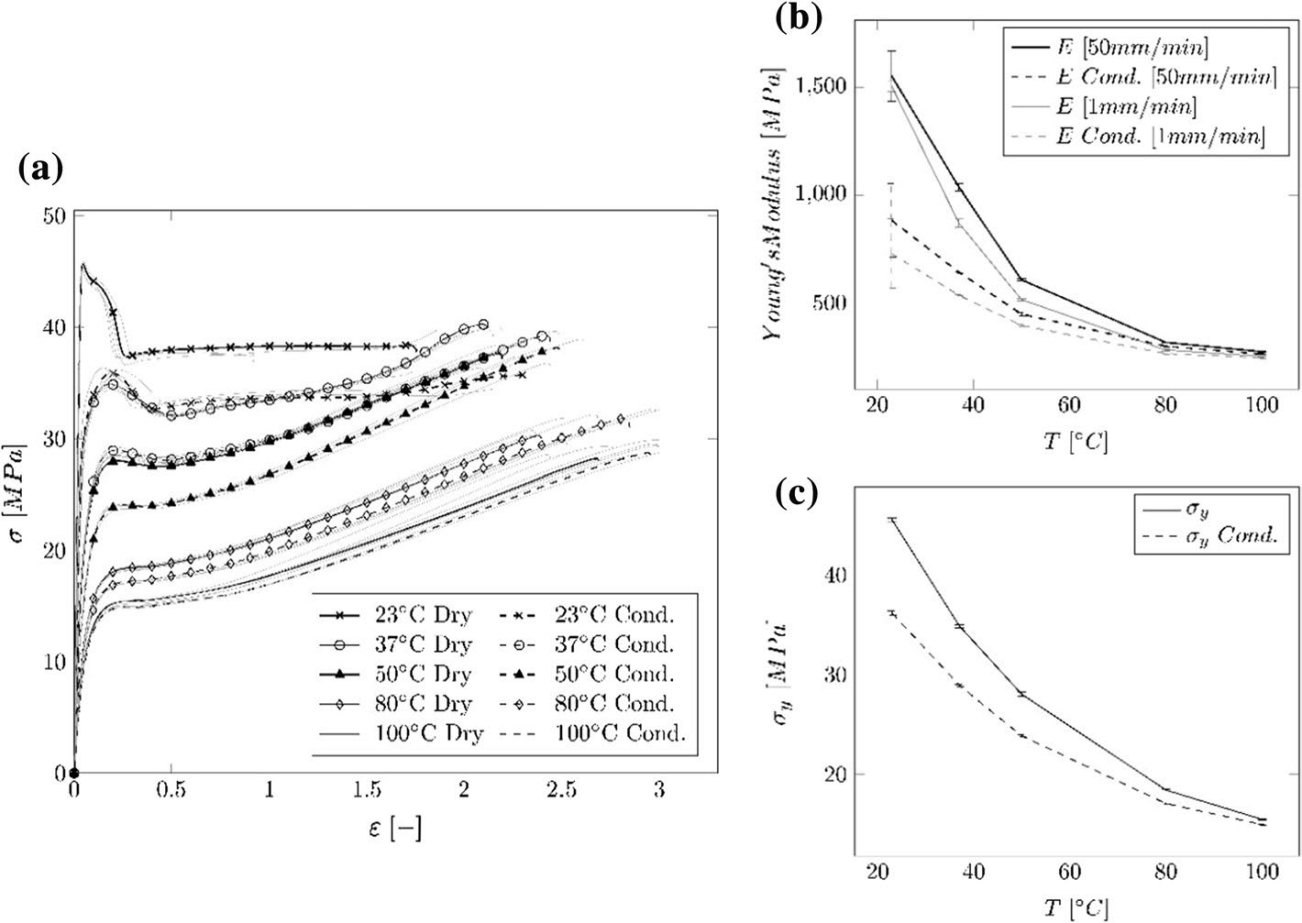
Results of the uniaxial tensile test of dog bone specimens at different temperatures and for conditioned and dry samples. **a** Nominal stress vs. strain curves. Black curves: sample lying in the center of the of the respective curve array, gray curves: scattering around the representative curve. **b** Median values of the Young’s Modulus over temperature with IQR for 1 mm/min and 50 mm/min. **c** Median values of ${\sigma }_{\mathrm{y}}$ over temperature with IQR

The water uptake of the specimens immersed in water was around 0.91% (interquartile range (IQR) = 0.15). The nominal stress–strain curves (cf. Fig. 1a), the Young’s Modulus against temperature (cf. Fig. 1b), as well as the yield stress against temperature (cf. Fig. 1c) obtained from the tensile test on the PA 12 dog bone specimens can be seen in Fig. 1. For visualization of the stress–strain curves, a representative sample, which lies in the center of the curve array, is selected for the respective specimen geometry, temperature level and polymer state (wet or conditioned). The other samples are displayed as grey lines. The respective median values and IQR of the Young’s Modulus $E$, yield stress ${\sigma }_{\mathrm{y}}$, drop ratio (DR) and Poisson's ratio $\nu$ obtained from the tensile tests are shown in Table 2. The specimens stored at ambient temperature and humidity (dry) show at 23 °C a median Young’s Modulus ${E}_{50 \mathrm{mm}/\mathrm{min}}=1553.49 \mathrm{MPa},\mathrm{ IQR}=233.1\mathrm{ MPa}$ (cf. Fig. 1b) and a yield point of ${\sigma }_{\mathrm{y}}=45.53 \mathrm{MPa},\mathrm{ IQR}= 0.4\mathrm{ MPa}$ (cf. Fig. 1c). At 100 °C the Young’s Modulus as well as the yield stress ${\sigma }_{y}$ are reduced to $250.62$ MPa, IQR = 1.48 MPa and $15.5 \mathrm{MPa},\mathrm{ IQR }= 0.1 \mathrm{MPa}$, respectively.

**Table 2:** Median values and IQR of the Young’s Modulus $E$, yield stress ${\sigma }_{\mathrm{y}}$ and DR of the tensile tests on dog bone specimens

Temp. [°C]	State	${E}_{1\mathrm{mm}/\mathrm{min}}$ [MPa] (IQR)	${E}_{50\mathrm{mm}/\mathrm{min}}$ [MPa] (IQR)	${\sigma }_{y}$ [MPa] (IQR)	$DR$ [%] (IQR)	${\nu }_{1\mathrm{mm}/\mathrm{min}}$ [−] (IQR)	${\nu }_{50\mathrm{mm}/\mathrm{min}}$ [−] (IQR)
23	Dry	1506.5 (51.4)	1553.5 (233.1)	45.5 (0.4)	82.8 (0.9)	0.47 (0.06)	0.37 (0.19)
	Cond	728.0 (161.3)	882.0 (341.5)	36.2 (0.4)	90.9 (0.9)	0.36 (0.14)	0.31 (0.11)
37	Dry	869.1 (36.6)	1036.3 (35.0)	34.9 (0.3)	91.9 (0.7)	0.44 (0.03)	0.44 (0.01)
	Cond	533.9 (6.2)	641.5 (5.4)	28.9 (0.2)	97.4 (0.3)	0.42 (0.01)	0.46 (0.01)
50	Dry	514.3 (5.5)	606.2 (11.1)	28.0 (0.4)	98.4 (0.8)	0.40 (0.03)	0.46 (0.01)
	Cond	393.6 (7.9)	446.4 (12.3)	23.8 (0.2)	–	0.41 (0.03)	0.46 (0.01)
80	Dry	280.0 (10.5)	315.2 (1.2)	18.4 (0.2)	–	0.35 (0.06)	0.47 (0.01)
	Cond	1263.6 (6.9)	297.6 (3.1)	17.1 (0.2)	–	0.32 (0.04)	0.47 (0.01)
100	Dry	250.6 (3.2)	272.1 (2.9)	15.5 (0.1)	–	0.43 (0.04)	0.47 (0.01)
	Cond	243.2 (4.7)	261.1 (3.9)	14.9 (0.1)	–	0.44 (0.07)	0.47 (0.01)

Both the dry as well as the conditioned samples show a horizontal stress course during neck propagation at 23 °C until the specimen fractures. At higher temperatures, a strain hardening can be observed after necking.

Through the conditioning at 23 °C, the DR is increased from 82.2% (23 °C, Dry) to 90.9%, meaning the force decreases less. For both the conditioned samples at 50 °C as well as for the samples tested at temperatures above 50 °C no DR is visible anymore.

The conditioned samples at 23 °C show a similar yield point and DR as the dry samples at 37 °C. The same is true for the conditioned samples at 37 °C and the dry samples at 50 °C. The Poisson’s ratio $\nu$ (cf. Table 2) at a test speed of 1 mm/min shows a high fluctuation over the temperature range for the dry and conditioned samples. At a test speed of 50 mm/min and 23 °C the Poisson’s ratio $\nu =0.37$, and $\nu =0.31$ for the dry and the conditioned specimens. At higher temperatures, $\nu$ becomes nearly constant at around 0.47 regardless of the conditioning.

Further graphs on material properties (${\sigma }_{\mathrm{p}}$, DR, ${\sigma }_{\mathrm{u}}$ and ${\varepsilon }_{\mathrm{u}})$ can be found in the appendix.

### Results of the tube tests

**Fig. 2:**
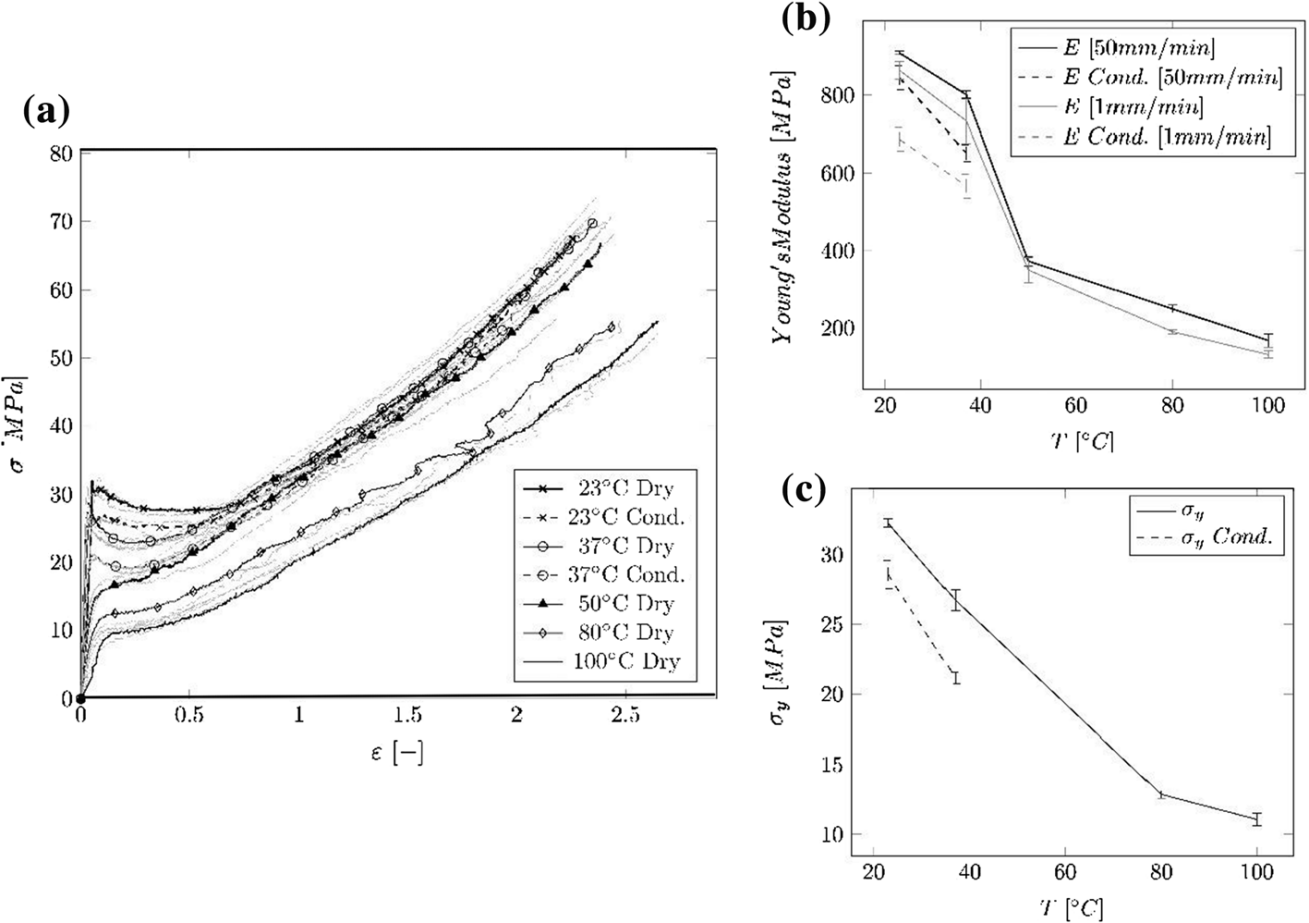
Results of the uniaxial tensile test on medical PA 12 tubes at different temperatures and for conditioned and dry samples. **a** Nominal stress vs. strain curves. Black curves: sample lying in the center of the of the respective curve array, gray curves: scattering around the representative curve. **b** Median values of the Young’s Modulus over temperature with width of the IQR. **c** Median values of ${\sigma }_{\mathrm{y}}$ over temperature with width of the IQR

The nominal stress–strain curves (cf. Fig. 2a), the Young’s Modulus against temperature (cf. Fig. 2b), as well as the yield stress against temperature (cf. Fig. 2c) obtained from the tensile test on the PA 12 tubes can be seen in Fig. 2. The respective median values and IQR of the Young’s Modulus $E$, yield stress ${\sigma }_{\mathrm{y}}$ and DR of the tensile tests are shown in Table 3. The tubes show a Young’s Modulus ${E}_{50\mathrm{mm}/\mathrm{min}}=908.7\mathrm{ MPa},\mathrm{ IQR}=8.54\mathrm{ MPa}$ and ${\sigma }_{y}=32.3\mathrm{ MPa},\mathrm{ IQR}=0.6\mathrm{ MPa}$ (cf. Fig. 2c) at 23 °C. The yield point is followed by a DR of 86.9%, IQR = 1.26% and 86.8%, IRQ = 2.8% for the dry and the conditioned specimens, respectively. After the neck stabilizes a horizontal stress course during neck propagation is visible followed by a strain hardening of the material until it fractures. At 37 °C the DR is reduced to 83.8%, IQR = 0.89%, indicating that the force decreases more than at 23 °C for the dry specimens and increases to 90.9%, IQR = 2.1% for the conditioned specimens. At temperatures above 37 °C no DR is visible anymore.

**Table 3:** Median values and IQR of the Young’s Modulus $E$, yield stress ${\sigma }_{\mathrm{y}}$ and DR of the tensile tests on tube specimens

Temperature [°C]	State	${E}_{1\mathrm{mm}/\mathrm{min }}$ [MPa] (IQR)	${E}_{50\mathrm{mm}/\mathrm{min}}$ [MPa] (IQR)	${\sigma }_{y}$ [MPa] (IQR)	$DR$ [%] (IQR)
23	Dry	863.9 (44.9)	908.7 (8.5)	32.3 (0.6)	86.9 (1.3)
	Cond	687.6 (58.9)	844.6 (30.7)	28.6 (2.0)	86.8 (2.8)
37	Dry	733.5 (113.5)	801.1 (19.2)	26.7 (1.5)	83.8 (1.8)
	Cond	567.3 (62.8)	651.9 (20.6)	21.2 (0.8)	90.9 (2.1)
50	Dry	349.9 (64.2)	372.8 (23.6)	–	–
80	Dry	190.1 (10.8)	249.5 (19.0)	12.9 (0.5)	–
100	Dry	131.2 (18.2)	167.9 (34.4)	11.1 (0.9)	–

**Fig. 3:**
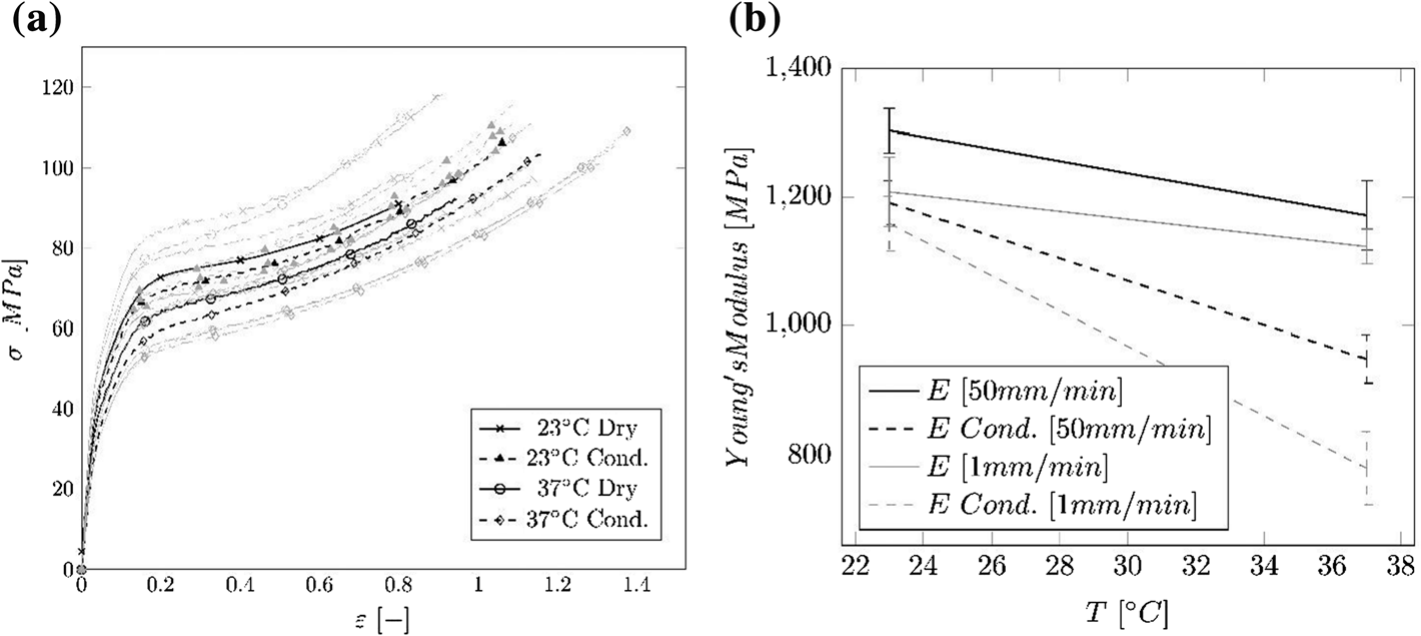
Results of the uniaxial tensile tests on necked PA 12 tubes at 23 °C and 37 °C for conditioned and dry samples. **a** Nominal stress vs. strain curves. Black curves: sample lying in the center of the of the respective curve array, gray curves: scattering around the representative curve. **b** Median values of the Young’s Modulus for 23 °C and 37 °C with width of the IQR

The nominal stress–strain curves (cf. Fig. 3a) and the Young’s Modulus against temperature (cf. Fig. 3b), obtained from the tensile test on the necked PA 12 tubes, can be seen in Fig. 3. Compared to the plain tubes, the necked specimens do not possess a yield point. Therefore, the proportional limit ${\sigma }_{p}$ is shown in addition to the Young’s Modulus $E$ in Table 4.

**Table 4:** Median values and IQR of the Young’s Modulus $E$ and proportional limit ${\sigma }_{\mathrm{p}}$ of the tensile tests on tube specimens

Temperature [°C]	State	${E}_{1\mathrm{mm}/\mathrm{min}}$ [MPa] (IQR)	${E}_{50\mathrm{mm}/\mathrm{min}}$ [MPa] (IQR)	${\sigma }_{p}$ [MPa] (IQR)
23	Dry	1209 (54.2)	1303.8 (69.7)	23.2 (0.9)
	Cond	1159.2 (85.7)	1191.6 (68.58)	19.53 (1.0)
37	Dry	1123.1 (54.7)	1172.0 (109.6)	17.68 (1.0)
	Cond	776.8 (114.8)	948.0 (75.6)	15.4 (1.1)

Even though there is a large scattering in the stress–strain curves of the necked tubes, an effect of conditioning or increased temperature is visible.

Further graphs on for the regular and the necked tubes (${\sigma }_{\mathrm{p}}$, DR, ${\sigma }_{\mathrm{u}}$ and ${\varepsilon }_{\mathrm{u}}$) can be found in the Appendix.

## Discussion

### General polymeric behavior

The dog bone specimens and the regular tubes show a typical behavior for semi-crystalline polymers, featuring (i) an elastic region with reversible deformations defined by the Young’s Modulus; (ii) yielding; (iii) necking with a local decrease in the cross-sectional area; (iv) cold drawing where the neck extents; (v) strain hardening where the chains are oriented parallel to the stretching direction, and (vi) fracture. When comparing the obtained Young’s Modulus from the dog bone test to the literature [8], the difference between the Young’s Modulus at 23 °C was + 7.6% and − 33.8% for the dry and conditioned and specimens, respectively. The yield stress ${\sigma }_{\mathrm{y}}$ was found to be + 1.2% and − 9.5% for the dry and conditioned samples, respectively. The highest mechanical strength and Young’s Moduli were found at the lowest temperature tested (23 °C). The higher the temperature, the higher the mobility of the chains, and therefore, the material starts to soften. Hence, mechanical properties like the Young’s Modulus, the yield point, and the proportional limit decrease.

By conditioning the samples in water, an additional softening of the material can be observed. This effect is also well known for polyamides [5] and can be explained by the water molecules forming weak bonds with the polar amide group. The interaction with the water increases the chain mobility and acts as a plasticizer, and as a result, the mechanical properties are reduced. In Table 5, the effect of the conditioning on the Young’s Modulus can be seen. For the regular and the necked tubes, the difference between dry and conditioned samples was less prominent. Therefore, it is assumed that the water absorption is increased when stored at ambient temperature and humidity. A possible explanation for this effect is the smaller wall thickness compared to the dog bone specimen, or the water uptake occurring on the inner and outer surface of the tube due to the hollow shape.

**Table 5:** Reduction of the Young’s Modulus of the conditioned samples compared to the dry samples for the dog bone, tube, and necked tube specimens at a test speed of 50 mm/min at each temperature level

Sample	23 °C	$37$ °C	$50 ^\circ \mathrm{C}$	80 °C	100 °C
Dog bone	− 51.7 [%]	− 38.6 [%]	− 23.4 [%]	− 5.9 [%]	− 2.9 [%]
Tubes	− 7.0 [%]	− 18.6 [%]	–	–	–
Necked Tubes	− 8.6 [%]	− 19.1 [%]	–	–	–

**Fig. 4:**
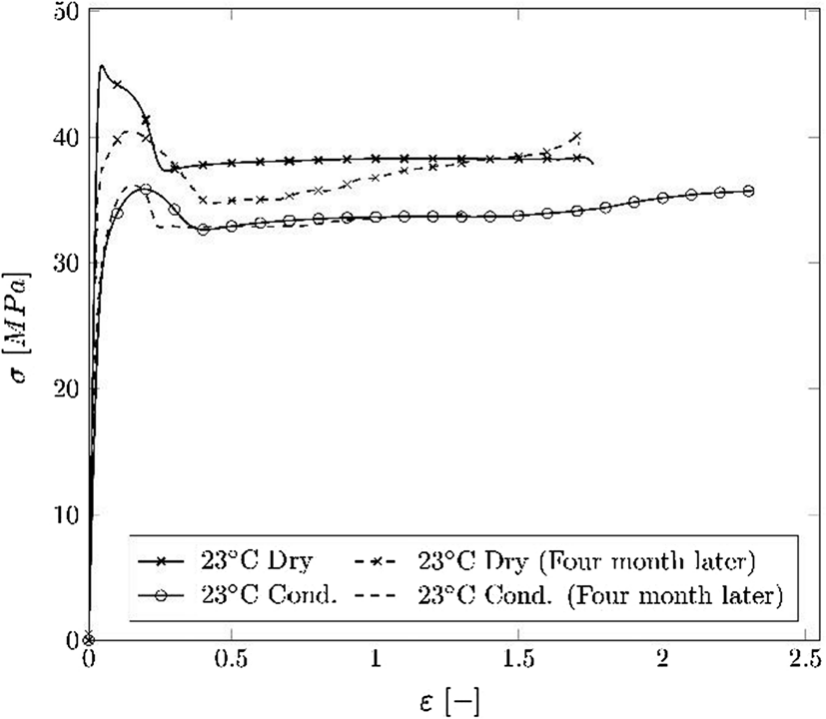
Nominal stress vs. strain curves of the dog bone specimen dry, conditioned, and stored at ambient climate over a period of 4 months

Based on those results, another test was performed on six dog bone specimens in which half of the specimens were conditioned in water, and the other half was stored at ambient climate over a period of another 4 months after the first test. The median result can be seen in Fig. 4 and shows that the water uptake is a time-dependent process which takes place more quickly in an aqueous environment.

There was found to be no measurable difference in the yield point between specimens conditioned in water for the defined 40 days and those conditioned for an additional 4 months. However, the samples stored at ambient temperature and humidity (23 °C and 50% r. H.) showed a reduction in yield stress after the same 4-month time period.

Strain rate dependency is a well-known effect in polymers [12]. In contrast to the temperature, the increase of the strain rate causes a stiffening of the material. The average increase of the Young’s Modulus that occurs during an increase in the tensile velocity from 1 to 50 mm/min can be seen in Table 6. This effect is not further investigated, as the focus of his work was set on the effects of temperature and specimen geometry.

**Table 6:** Average increase of the Young’s Modulus for the dog bone, tube and necked tube samples at a test speed of 50 mm/min compared to a test speed 1 mm/min

Sample	Wet	$\mathrm{Cond}.$
Dog bone	+ 12.3 [%]	+ 15.0 [%]
Tubes	+ 16.0 [%]	+ 18.9 [%]
Necked Tubes	+ 6.1 [%]	+ 12.4 [%]

### Comparison between the specimen groups

Unlike the regular tubes (cf. Fig. 2a), the dog bone specimens (cf. Fig. 1a) showed no strain hardening at 23 °C. At elevated temperatures, both types of specimens showed a similar behavior. At 23 °C, the measured Young’s Modulus and the yield point for the dry tubes were about 41.5% and 29.2% less than compared to the dry dog bone specimens. The difference in these values could be due to effects of the clamping, changes in the material, and/ or changes of the deforming specimen structure and the corresponding specimen dimension (rectangular vs. hollow profile). The deformation of the hollow tube structure may have introduced a different elastic deformation compared to the rectangular cross section. A possible explanation is that there is no material accumulation in the center of the tubes and that chain molecules are less confined. Hence, the chain molecules have a higher mobility, which in return softens the material. Furthermore, due to the small cross section of the tubes, variations in wall-thickness may have a greater influence on the overall mechanical properties. However, the regular tubes (cf. Fig. 2a) show a small scatter of the stress–strain curves, which means that either the variation of the wall-thickness over the length is comparable for all tube samples, or that the variation is small enough to not have an influence on the mechanical properties. When comparing the ultimate strength ${\sigma }_{\mathrm{u}}$ of the regular tubes (cf. Fig. 2a) and the dog bone specimens (cf. Fig. 1a), it can be seen that the tubes demonstrate a higher ${\sigma }_{\mathrm{u}}$. A possible size influence could be a reason for this effect. The cross section of the dog bone specimens as well as the initial gauge length are larger compared to the tubes. The probability of failure occurring in the event of a material defect is greater with large specimens. For a detailed understanding of the size effect, dog bone specimen with a similar cross section and length scale would be necessary. This analysis goes beyond the scope of this manuscript.

By necking the tubes, the Young’s Modulus is increased by about 43.5% and 41.1% for the dry and the conditioned specimens, respectively. Roesler [6] suggested that by alignment of chain molecules, the crystallinity can be increased. However, the DSC measurements showed that there is no difference in crystallization between the specimen groups. Therefore, it can be derived that the strengthening of the material is rather based on the prior stretching and orientation of the chain molecules parallel to the load direction, than on the crystallinity. This dependency can also be seen when comparing the ultimate strain (cf. Fig. 3a) of the necked tubes with that of the regular tubes (cf. Fig. 2a). The strain measured in the necked tubes is about 50% less than the strain received from the regular tube measurement. This shows that the necked tubes already possess some induced deformation and damage of the chain network.

There exist some simple models [6, 13, 14], that describe the behavior of semi-crystalline polymers under tension. Since the DSC analysis is not able to give insights into the molecular chain orientation, further investigations would be necessary. Other methods like polarized absorption measurements or transmission electron microscopy (TEM) could give more detailed insights to prove conformity with these models.

### PTCA balloon catheters

Even if PA 12 has the lowest amide group concentration, and therefore a low moisture absorption, the influence of the water uptake on the mechanical properties cannot be neglected and must be considered in the development process of products. Especially the burst-pressure as well as the tensile properties of catheters could vary significantly, when exposed to elevated humidity and temperatures. The mechanical properties of the tubes are reduced compared to the standardized dog bone specimen. This could lead to an overestimation of the strength during the design process (e.g., in finite element (FE) simulations). Therefore, it is recommended to use catheter-specific properties. Predefined forming steps like necking allow the mechanical properties to be specifically adapted. Besides the increase of the mechanical properties in longitudinal direction due to necking, also an increase in pressure-resistance could be observed (see Appendix, Fig. 12). This allows, for example, a reduction of the cross section without sacrificing performance due to the increased strength.

The forming of the balloon itself usually requires several process steps [10]. In the first step a parison, where both ends of a PA 12 tube are necked, is manufactured. Afterwards, a balloon is formed from the parison in a stretch–blow–molding process under pressure and temperature. To further reduce the wall-thickness a secondary stretch is applied to the balloon. Due to the differences in the necking and stretching process, the obtained mechanical properties for the catheter shaft will vary from those of the balloon. Not only is there a secondary stretching, but also an increased stretching in radial direction.

In order to open calcified stenoses high pressures are required. To avoid stent malapposition or vessel injuries, the balloon is required to change little in volume with increasing pressure. Since the necking and stretching influences the pressure resistance and the stiffness, this process is required for the manufacturing of such so called non-compliant balloons. If a balloon does not dilate properly during an intervention it might become necessary to inflate the balloon multiple times. Therefore, not only the quasistatic behavior, but also the fatigue behavior is of importance. Due to the thin wall thickness and the large diameter of the balloon is the weakest part of the catheter regarding burst resistance. Since our study focuses on the catheter shaft and the tube prior to balloon forming this effect is neglected in this study.

### Limitations

For the dog bone specimens, the scattering of the Young's Modulus is the highest at 23 °C (${\mathrm{IQR}}_{\mathrm{dry}}=233.1$ MPa and ${\mathrm{IQR}}_{\mathrm{cond}.}=341.5$ MPa). One explanation for this larger scattering are uncertainties present in the video extensometer strain measurement. Such uncertainties are assumed to be caused by slight rotations of the measuring marks on the sample surface, since the scattering was not visible in the measured strain from the traverse of the tensile testing machine. This effect was, however, only observed at 23 °C and does not influence the measured force.

Our method only determined an average cross section for the tubular specimens, and therefore, the wall-thickness variation over the length remains unknown. For the regular tubes manufactured in a controlled environment, the influence of the wall-thickness variation seems to be low, whereas this effect can probably not be neglected for the necked tubes, as they show a notably larger scattering. A possible explanation for this effect could be the manual manufacturing process of the tube forming. In this process, no constant velocity could be guaranteed, which in return can affect the properties in longitudinal direction of the sample as well as the wall-thicknesses.

The difference between the dry and conditioned dog bone samples becomes smaller when increasing the temperature. This is not necessarily a temperature-dependent effect, but could also happen due to an already incipient drying when warming up the samples. Due to this fact, no conditioned tubes were tested above 37 °C. A potential anisotropy, induced during the manufacturing of the tubes, was not investigated in this study.

## Conclusion

PA 12 is a versatile material whose properties can be adapted in different ways. However, the mechanical properties can vary a lot between specimen geometries, manufacturing processes, and prior forming steps, such as necking. Necking is important for improving material strength, however, the process needs to be well-defined and controlled, to reduce the variation and overall performance of the final product. In our study, we showed a softening of PA 12 with increasing temperature. The Young’s Modulus as well as the yield stress is reduced by around 17–25% when increasing the temperature from 23 to 37 °C. Accounting for the targeted operating temperature during the design process is therefore of key importance and increases the safety and performance of future PTCA catheters.

## Methods

In this work, uniaxial tensile tests were carried out on standardized dog bone specimens and extruded PA 12 tubes used in the manufacturing of PTCA balloon catheters. The tensile tests were conducted at five different temperatures ${T}_{\mathrm{i}}$, ranging between 23 and 100 °C. At each temperature level, 10 specimens were tested, whereby half the 10 specimens were conditioned in water for 40 days at 23 °C, and the other half was stored under ambient laboratory conditions (approx. 23 °C and 50% relative humidity). In a previous study it was found that the influence of a solution with physiological buffers, such as a 0.9% saline solution, over the duration of an intervention (approximately 1–2 min) is neglectable (see Appendix) and therefore not considered in the further conditioning.

In addition, 20 PA 12 tubes were processed by a necking process in which the tube is pulled through a nozzle that reduces the tube dimensions. The necked tubes were also subjected to a tensile load at 23 °C and 37 °C, respectively. The crystallinity, the glass transition temperature ${T}_{\mathrm{G}}$, and the melting temperature ${T}_{\mathrm{m}}$ were assessed for each specimen geometry (dog bone, tube, and necked tube) by DSC of type DSC 3 + (Mettler-Toledo AG, Switzerland) on one sample. The DSC analyses were performed in a nitrogen environment during one cycle with a heating/cooling ramp of 10 °C/min between 0 and 300 °C.

### Experimental setup for the dog bone specimens

The specimens (ISO 527-2, Type 1A, EMS-CHEMIE AG, Switzerland) were fabricated by injection molding of Grilamid® L25 granules. Their initial cross section ${A}_{0}$ was roughly $40 {\mathrm{mm}}^{2}$. For each specimen, the width and thickness of the middle section were measured with a caliper.

In order to evaluate the water absorption (cf. Eq. 1), the mass of the specimen was measured with a scale of type MC21S (Sartorius AG, Goettingen, Germany) before and after the immersion, ${m}_{\mathrm{amb}}$ and ${m}_{\mathrm{wet}}$, respectively.

1 $$\mathrm{Increase in weight}=\frac{{m}_{\mathrm{wet}}-{m}_{\mathrm{amb}}}{{m}_{\mathrm{amb}}}\bullet 100 [\mathrm{\%}]$$

**Fig. 5:**
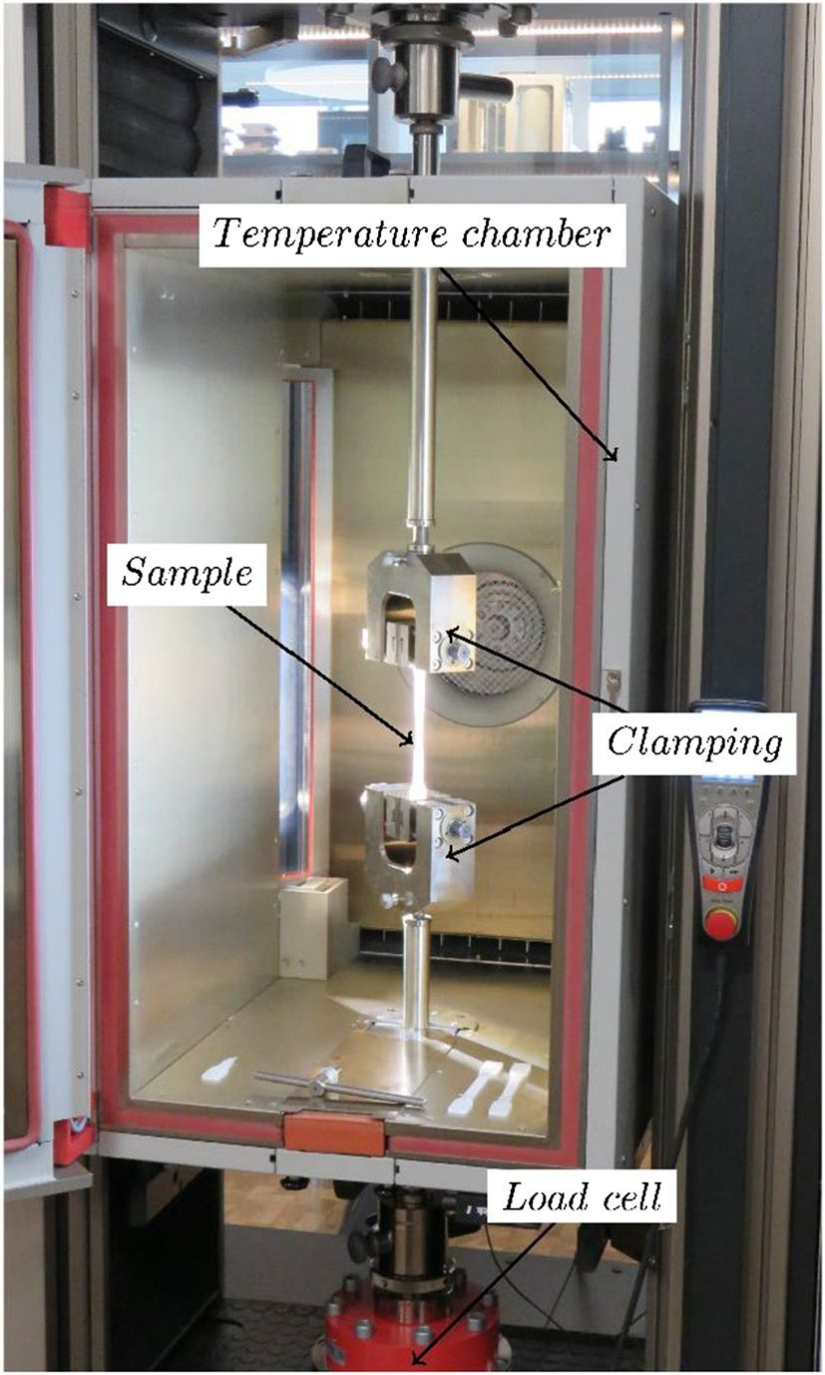
Setup of the Zwick Z250 with environmental chamber and clamped specimen

The uniaxial tensile tests were carried out on a material testing machine Zwick Z250 (ZwickRoell GmbH & Co. KG, Ulm, Germany) with a temperature chamber (± 1 °C) (cf. Fig. 5). The forces were measured in [N] with a 5-kN load cell. Local deformations in transverse and longitudinal direction were measured using the Zwick VideoXtens video-extensometer with glued-on measuring marks at an initial gauge length of ${L}_{0}=75 \mathrm{mm}$.

The tests were performed based on ISO 527–2. The ISO defines a test speed of $1 \mathrm{mm}/\mathrm{min}$ for measuring the Young's Modulus $E$ and the Poisson's ratio ν and a test speed of $50 \mathrm{mm}/\mathrm{min}$ for the mechanical properties until fracture. Therefore, at 0.6% strain, the specimen was relieved and re-loaded with a test speed of 50 mm/min until breakage. The change in test speed is required on one hand, to allow for a good accuracy of the Young’s Modulus and on the other hand limits the effect of a time depended behavior onto the mechanical properties.

### Necking of tubes

Necking is a process commonly used for balloon forming. It is done prior to the stretch–blow molding to reduce the dimensions of the catheter and to control the location of the balloon [15]. During the necking process, the polymeric tube is heated locally and stretched in axial direction. The axial stretch causes an alignment of the molecule chains within the tube. As explained by Roesler [6], the strength of semi-crystalline polymers can be increased either by a rise in crystallinity or by orientation of chain molecules (stress-induced crystallization). Therefore, the necking process is expected to influence the mechanical strength of the PA 12.

**Fig. 6:**
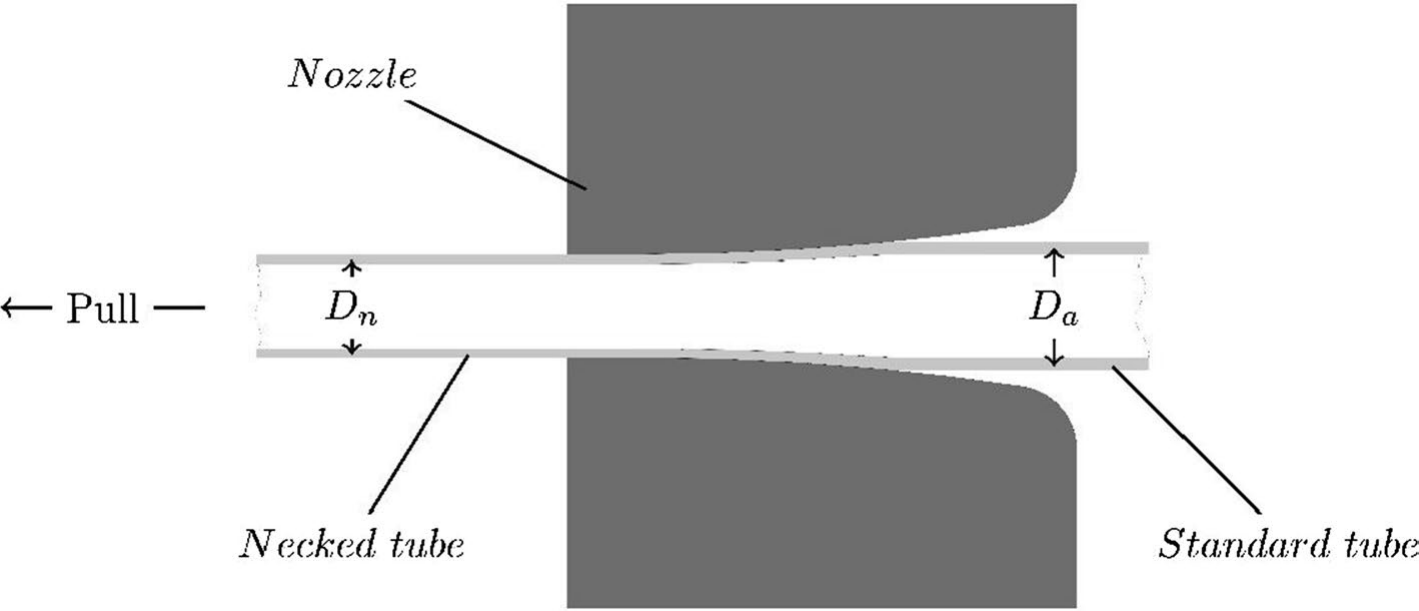
Necking process with an unheated nozzle with outer diameter ${D}_{\mathrm{a}}=0.92 \mathrm{mm}$, before and ${D}_{\mathrm{n}}=0.82 \mathrm{mm}$ after necking

Standard single-lumen monolayer Grilamid® L25 PA 12 tubes, with an outer diameter ${D}_{\mathrm{a}}$ of 0.92 mm, for catheter manufacturing, were delivered by Putnam Plastics, USA. In the necking process used during this study, the PA 12 tubes were manually pulled through an unheated tapered nozzle (cf. Fig. 6). For achieving better control of the inner diameter, a calibration wire was inserted, and in order to relieve inner tensions the necked samples were tempered for 60 min at 50 °C.

### Experimental setup for the tubes

Since conventional measurements with a caliper impose a risk to harm the tube, the cross section was instead determined by measuring the mass $m$ and length $L$ of the samples (cf. Eq. 2). The density of the material is specified by the manufacturer ($\rho =1010\frac{kg}{{m}^{3}}$):

2 $$A= \frac{m}{\rho \bullet L} \left[{\mathrm{mm}}^{3}\right].$$

The uniaxial tensile tests of the PA 12 tubes were conducted on a Zwick Z010 (ZwickRoell GmbH & Co. KG, Ulm, Germany) using a temperature chamber. Various clamping devices, such as pneumatic grips, with pyramid or rubber jaws, as well as capstan grips which are usually used for yarn testing, were evaluated to find the most suitable device for preventing any sliding of the tubes during testing. The rubber jaws proved to be the best solution against slippage, and simultaneously, allowed for testing of short samples with ${L}_{0}=45 \mathrm{mm}$ and measuring failure in the limited travel range of the machine. In order to stabilize the lumen from collapsing between the clamps, wire inserts were implemented. Four markers were applied at 10 mm distances onto the specimen to measure local deformations with a video extensometer. Whenever fracture occurred close or inside the clamps, test results were discarded, and the experiment was repeated.

As in the tests of the dog bone specimens, a test speed of $1 \mathrm{mm}/\mathrm{min}$ was defined for the start of the measurement. After a strain of $0.3\%$ was reached, the test speed was increased to $50 \mathrm{mm}/\mathrm{min}.$

The tensile tests were carried out at 23 °C, 37 °C, 50 °C, 80 °C and 100 °C for the standard tubes and at 23 °C and 37 °C for necked as well as conditioned tubes.

### Material properties

For each material parameter, the median as well as the IQR were determined. The characteristic nominal stress $$(\sigma )$$ (cf. Eq. 3) vs. longitudinal strain $$({\varepsilon }_{\mathrm{l}})$$ (cf. Eq. 4) curves are obtained by converting the measured force $$F$$ and video-extensometer displacement $$(\Delta L)$$ by means of the initial cross section ($${A}_{0})$$ and the initial gauge length $$({L}_{0})$$, respectively:

3 $$\sigma = \frac{F}{{A}_{0}} \left[MPa\right],$$

4 $${\varepsilon }_{l}= \frac{\Delta L}{{L}_{0}} \left[-\right].$$

The Poisson's ratio $(\nu )$ describes the ratio of transverse contraction $({\varepsilon }_{\mathrm{t}})$ and longitudinal extension $({\upvarepsilon }_{\mathrm{l}})$ (cf. Eq. 5) (ASTM D638). For most materials under tension, $\nu$ lies in a range of 0 to 0.5, whereas 0.5 is for incompressible materials (e.g., rubber). The Poisson’s ratio $\nu$ was obtained from the slope of a linear fit (Matlab R2019a, MathWorks, Massachusetts, United States) between ${\varepsilon }_{l}$ = 0.25–0.6% for 1 mm/min and ${\varepsilon }_{\mathrm{l}}=0.6-0.95$ for 50 mm/min for the measurements of the dog bone sample:

5 $$\nu = \frac{\Delta {\varepsilon }_{t}}{\Delta {\varepsilon }_{l}} \left[-\right].$$

For each specimen, the Young's Modulus $E$ (cf. Eq. 6) was obtained from the slope of a linear fit of $\sigma$ and ${\varepsilon }_{\mathrm{l}}$ between ${\varepsilon }_{\mathrm{l}}=0.05-0.3 \%$ for 1 mm/min and ${\varepsilon }_{\mathrm{l}}=0.6-0.8 \%$ for 50 mm/min:

6 $$E= -\frac{\Delta \sigma }{\Delta \varepsilon } \left[MPa\right].$$

**Fig. 7:**
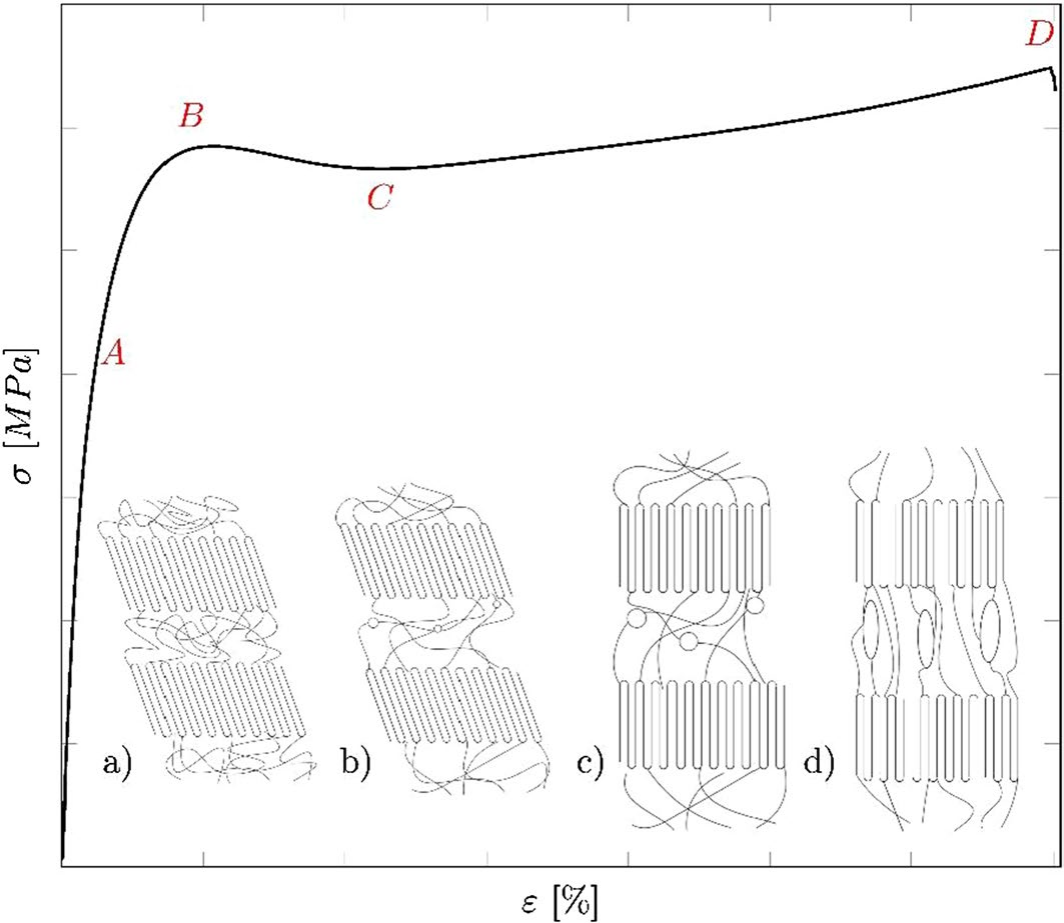
Exemplary nominal stress–strain curve of a semi-crystalline thermoplastic material. A = proportional limit; B = yield point; C = neck stabilization; D = fracture. **a** Undeformed chains, **b** elongated amorphous region and start of void formation, **c** disentanglement of amorphous chains, alignment of crystalline region and enlargement of voids, **d** formation of fibers (after [6, 14])

**Fig. 12:**
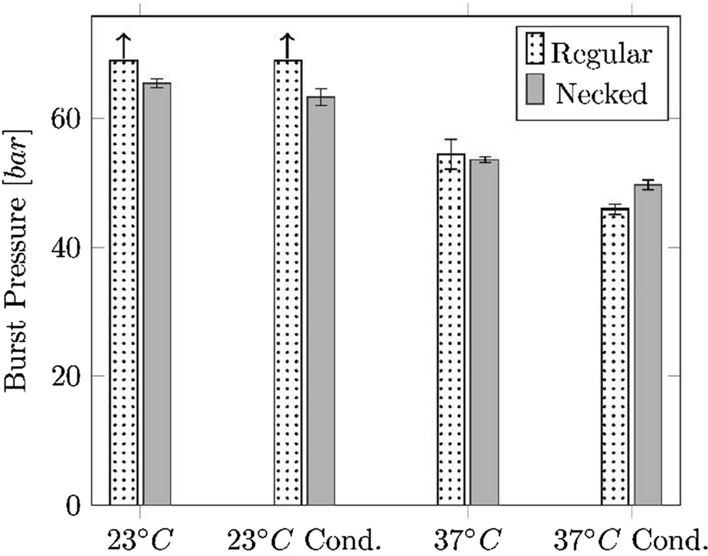
Median burst-pressure with IQR for regular and necked tubes at 23 °C and 37 °C for dry and conditioned specimen

The proportional limit is specified as a deviation from the proportionality of the stress–strain curve and marked by ${\sigma }_{\mathrm{p}}$ and ${\varepsilon }_{\mathrm{p}}$, respectively. To evaluate the proportional limit an offset-criterion is used. A line with a slope equal to the samples Young's Modulus is shifted by a strain-offset of 0.05%. The intercept between the nominal stress–strain curve and the offset-line defines the proportional limit A (cf. Fig. 7). The point B defines the yield point, which describes the stress ${\sigma }_{\mathrm{y}}$ and elongation ${\varepsilon }_{\mathrm{y}}$ after which the specimen starts to neck, i.e., the cross-sectional area decreases. The drop ratio (DR) is defined as the ratio between the stress at the point of neck stabilization (*C*) and the yield point (*B*) ($\mathrm{DR}=C/ B$). Point D is defined as the ultimate tensile stress ${\sigma }_{\mathrm{u}}$ and elongation at fracture ${\varepsilon }_{\mathrm{u}}$, respectively.

The complex chain alignment during the tensile test has been explained by a simple model. Since the bond strength in the crystalline region is higher than in the amorphous region, the amorphous molecules begin to lengthen (elastic region) and to untangle first. At larger strains (yield point), micro- and nano-voids start to form within the amorphous region, while the crystalline domains begin to orientate into the stress direction. At large strains the crystalline regions start to separate from each other, form blocks and finally form microfibrils [6, 13, 14].

## Figures and Tables

**Fig. 8:**
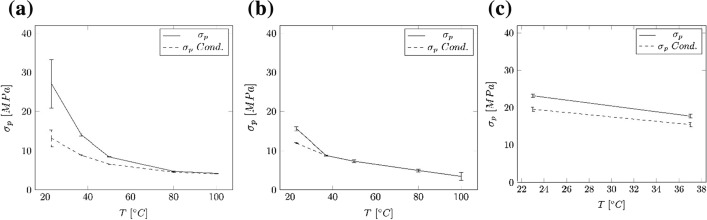
Median stress of the proportional limit ${\sigma }_{\mathrm{p}}$ with IQR over temperature for **a** the dog bone specimen, **b** the regular tubes, and **c** the necked tubes

**Fig. 9:**
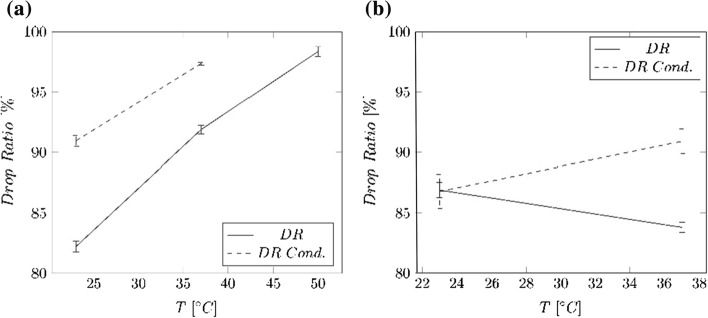
Median drop ratio DR with IQR over temperature for **a** the dog bone specimen, **b** the regular tubes, and **c** the necked tubes

**Fig. 10:**
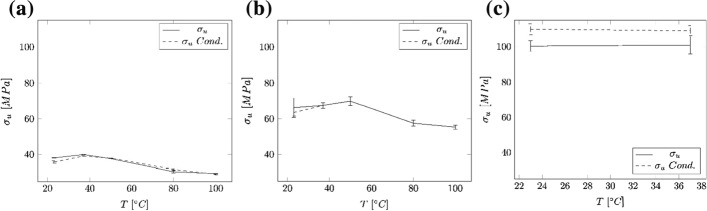
Median ultimate strength ${\sigma }_{\mathrm{u}}$ with IQR over temperature for **a** the dog bone specimen, **b** the regular tubes, and **c** the necked tubes

**Fig. 11:**
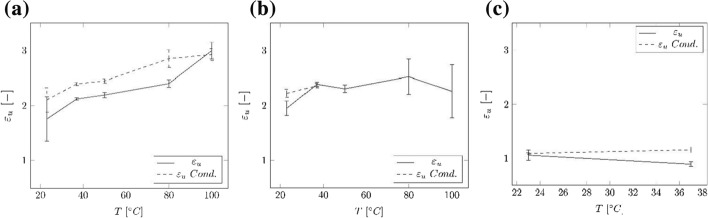
Median ultimate strain ${\varepsilon }_{\mathrm{u}}$ with IQR over temperature for **a** the dog bone specimen, **b** the regular tubes, and **c** the necked tubes

**Fig. 13:**
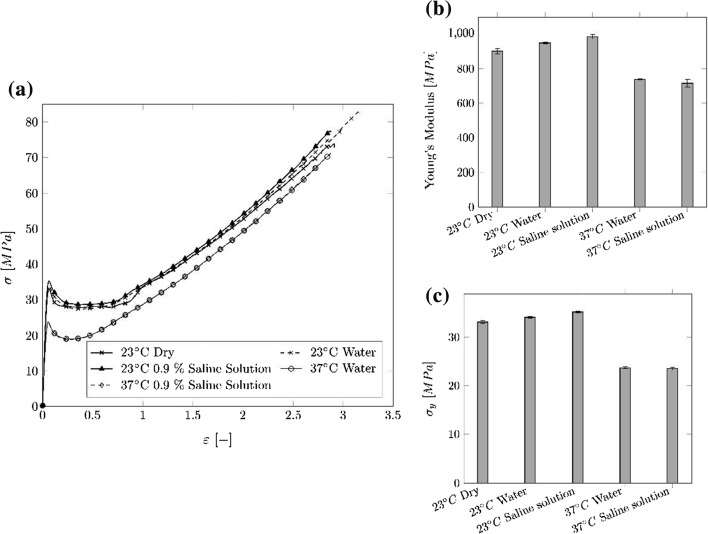
Results of the uniaxial tensile tests on necked PA12 tubes at 23 °C and 37 °C for specimens tested without water, in water, in a saline solution. **a** Nominal stress vs. strain curves. Sample lying in the center of the of the respective curve array. **b** Median values of the Young’s Modulus for 23 °C and 37 °C with width of the IQR. **c** Median values of ${\sigma }_{\mathrm{y}}$ with width of the IQR

## Data Availability

The datasets used and/or analyzed during the current study are available from the corresponding author on reasonable request.

## Appendix

### Mechanical properties depended on the temperature:

Proportional limit (cf. Fig. 8).

Drop ratio (cf. Fig. 9).

Ultimate strength (cf. Fig. 10)

Ultimate strain (cf. Fig. 11).

### Pressure resistance

In addition to the tensile tests 10 regular and 10 necked PA12 tubes were pressurized by a Crescent hydraulic Burst and Leak Tester 1000 (HBLT) (Crescent Design Inc., San Diego, USA). During these tests, the tubes are pressurized in a water bath with 23 °C and 37 °C from 0 to 69 bar.

The results of the pressure resistance test are depicted in Fig. 12. For the regular tubes at 23 °C no burst could be obtained in the operating range of the HBLT. The dry and conditioned necked tubes at 23 °C burst at 65.41 ± 0.7 bar and 63.3 ± 1.3 bar, respectively. At 37 °C a reduction of the burst-pressure was visible for the regular as well as the necked specimen. The dry and conditioned regular tubes burst at 54.4 ± 2.3 and 45.9 ± 0.7, respectively. The dry and conditioned necked tubes burst at 53.5 ± 0.4 and 49.6 ± 0.8, respectively.

By considering the wall-thickness, which is reduced by around 30% during the necking procedure, it was found, that the pressure resistance is increased by around 14–19% at 23 °C and 26–32% after the necking process.

### Influence of saline solution

During an intervention a balloon catheter gets in contact with blood. The application duration for such balloon catheters is around 1–2 min. In order to assess the influence of blood onto the polymeric material further uniaxial tensile tests have been carried out on a Shimadzu AGS-X 10 kN (Shimadzu Corporation, Kyoto, Japan) with a 200 N load cell analogous to the tests described in the methods section. Six specimens were tested either in a water bath filled with plain water or with a 0.9% saline solution at 23 °C and 37 °C. As a reference, another measurement has been carried out without water. Prior to the test begin, the specimens were kept in the water bath for 2 min to mimic the interventional duration.

The results of this test are shown in Fig. 13. At 23 °C a slight increase of approximately 3% of the Young’s Modulus (Fig. 13b) and the yield point (Fig. 13c) in saline solution becomes visible. At 37 °C the Young’s Modulus is reduced by − 3% and the yield point remains the same for the specimens in saline solution compared to those in water. The measurement at 23 °C without water is in a good agreement with the tested dry specimen (${E}_{50\mathrm{mm}/\mathrm{min}}=901.2 \mathrm{MPa},\mathrm{ IQR}=30.4\mathrm{ MPa}$ and ${\sigma }_{\mathrm{y}}=33.1\mathrm{ MPa},\mathrm{ IQR}=0.3\mathrm{ MPa}$) (see “Results”).

Based on those tests it can be concluded that the effect of a saline solution over the duration of an intervention is neglectable. Whereas the effect of 37 °C body temperature cannot be neglected due to the reduction of the mechanical properties.

## Abbreviations

PTCA: Percutaneous transluminal coronary angioplasty
PA: Polyamide
PET: Polyethylene terephthalate
PEBA: Polyether block amide
DSC: Differential scanning calorimetry
IQR: Interquartile range
DR: Drop ratio
TEM: Transmission electron microscopy
FE: Finite element
